# Solely MRI-Guided Cardiac Catheterization for Assessment of Pulmonary Hypertension in a Pregnant Lady with Undiagnosed Congenital Heart Disease

**DOI:** 10.1155/2020/3072869

**Published:** 2020-05-26

**Authors:** Aphrodite Tzifa, Ioannis Polymerou, Dimitra Loggitsi

**Affiliations:** ^1^Department of Congenital Heart Disease, Mitera Children's Hospital, Athens, Greece; ^2^Biomedical Engineering & Clinical Sciences, King's College London, UK; ^3^Department of Radiology, Mitera Children's Hospital, Athens, Greece

## Abstract

Pregnancy in women with complex congenital heart disease (CHD) can be poorly tolerated. Amongst pregnant women with CHD and pulmonary hypertension (PH), the mortality rate can be as high as 30%. Cardiac catheterization procedures for assessment of haemodynamics and pulmonary vascular resistance (PVR) are often required in this patient population for risk stratification. However, during the first few weeks of pregnancy, this should better be avoided due to the known adverse effects of the ionizing radiation to the immature fetus. In this setting, a solely MRI-guided catheterization may present as a better alternative.

## 1. Introduction

Persistent exposure of the pulmonary vasculature to increased blood pressure or blood flow from a systemic to pulmonary shunt can lead to pulmonary arterial hypertension (PAH) which is classified in Group 1 of the PH clinical classification. Some of these shunts can go unrecognized for years. In a retrospective cohort study on the prevalence of PAH in adults with a cardiac septal defect, PH was present in 28% of all patients or in 34% of patients with an open ASD and in 28% of patients with an open VSD and in 12% and 13% of patients with a closed defect, respectively [1].

Pregnant women with pulmonary hypertension have a high mortality and morbidity rate. Careful assessment of haemodynamics is crucial for their consultation with regard to pregnancy planning. However, on occasion, the diagnosis of pulmonary hypertension in the setting of congenital heart disease may be made for the first time during pregnancy. Risk stratification in such cases may require a cardiac catheterization procedure that normally exposes the woman and her fetus to ionizing radiation. We report on the case of a pregnant woman with newly diagnosed complex congenital heart disease and pulmonary hypertension who underwent solely magnetic resonance imaging- (MRI-) guided catheterization using real-time interactive imaging in order to ascertain anatomy, as well as haemodynamics and pulmonary vascular resistance, due to her wish to maintain pregnancy. To our knowledge, this is the first reported case of solely MRI-guided cardiac catheterization in pregnancy, expanding further the potentials of magnetic resonance imaging in cardiovascular disease.

## 2. Case Report

A 33-year-old woman presented with newly diagnosed pulmonary hypertension (PH) and significantly dilated right heart structures. She had been investigated in childhood for situs inversus abdominalis, but no definitive cardiac diagnosis was made. At presentation, she was 10 weeks pregnant and completely asymptomatic. Electrocardiogram showed negative p waves in lead II. Transthoracic echocardiography showed a large atrial septal defect with exclusive left to right shunt and severely dilated right ventricle with tricuspid valve velocity of 5 m/sec. The hepatic veins were imaged to drain directly into the right-sided atrium with no inferior vena cava flow seen; hence, the suspicion of left atrial isomerism was raised.

Due to evidence of pulmonary hypertension and her wish to maintain pregnancy, a cardiac catheterization procedure was advised, to assess the pulmonary arterial (PA) pressures and PVR. To avoid fluoroscopy at this early stage of pregnancy, we planned to perform a solely MRI-guided right heart catheterization, with which accurate PVR as well as the rest of the anatomy could be ascertained. The procedure was performed on the 15^th^ week of pregnancy in a High-Field Open MR Scanner (1.0T, Panorama, Philips). The following sequences were used: interactive planning for real-time imaging (TR-TE shortest, Act. TR/TE (ms) = 3.1/1.56, flip angle 45°), phase contrast flow sequences for Q flow measurements (TR-TE shortest, Act. TR/TE (ms) = 5.7/3.5, flip angle 15°), BTFE sequences for cine imaging (TR-TE shortest, Act. TR/TE (ms) = 5.1/2.1, flip angle 75°), and T1-weighted black blood sequences for anatomical imaging (TR = 667 ms,TE = 37 ms, flip angle 90°).

**Figure 1:**
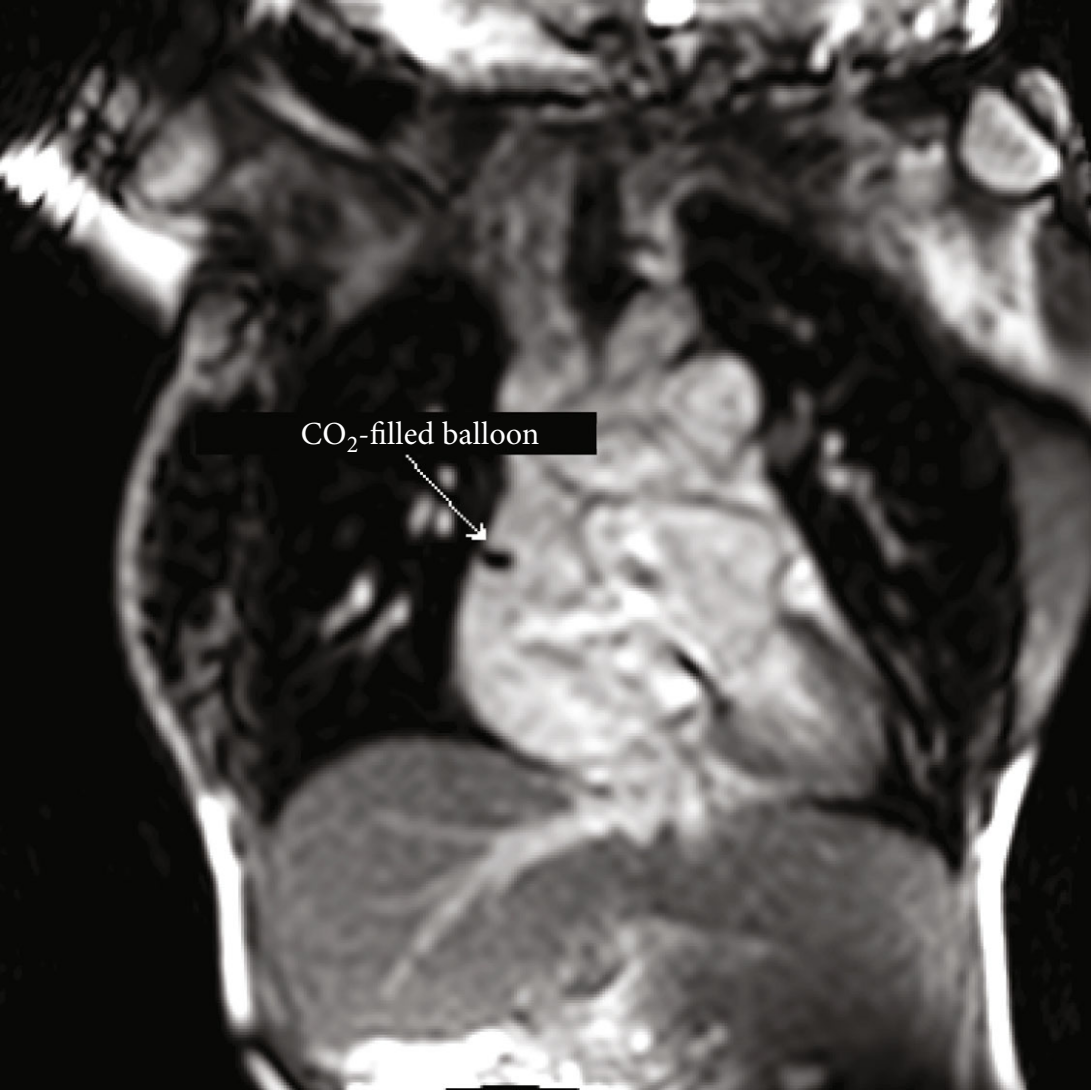
The balloon of the wedge catheter appears as a black circle in the white blood pool in the right-sided atrium. The hepatic veins appear to drain directly to the atrium.

The catheterization took place through the internal jugular vein, due to IVC interruption. An 8 French sheath was inserted into the right internal jugular vein under local anesthetic. The patient was transferred to the magnetic resonance suite where baseline real-time interactive imaging was initially performed. This confirmed IVC interruption with azygos continuation to the superior vena cava. The patient's hepatic veins drained directly to the right-sided atrium (Figure 1) whilst the liver extended from right to left throughout the upper abdomen. Polysplenia was also noted, and therefore, the diagnosis of heterotaxy/left atrial isomerism with an atrial septal defect was confirmed.

**Figure 2:**
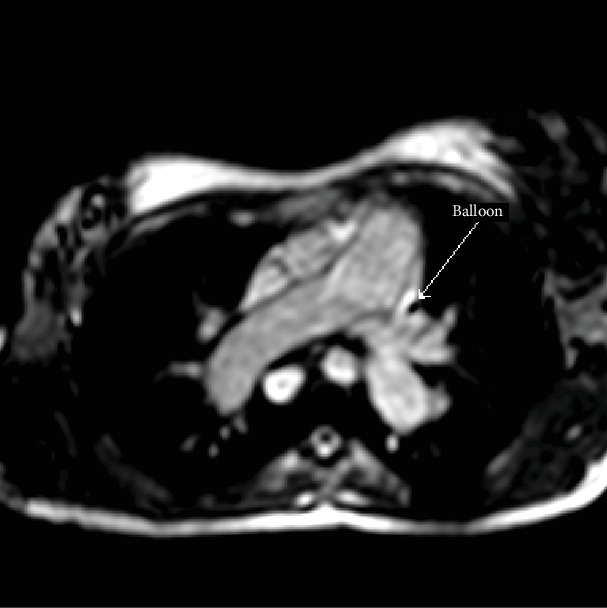
The CO_2_-filled balloon appears black in the SSFP images. The image shows the CO_2_-filled balloon of the wedge catheter in the left pulmonary artery.

**Figure 3:**
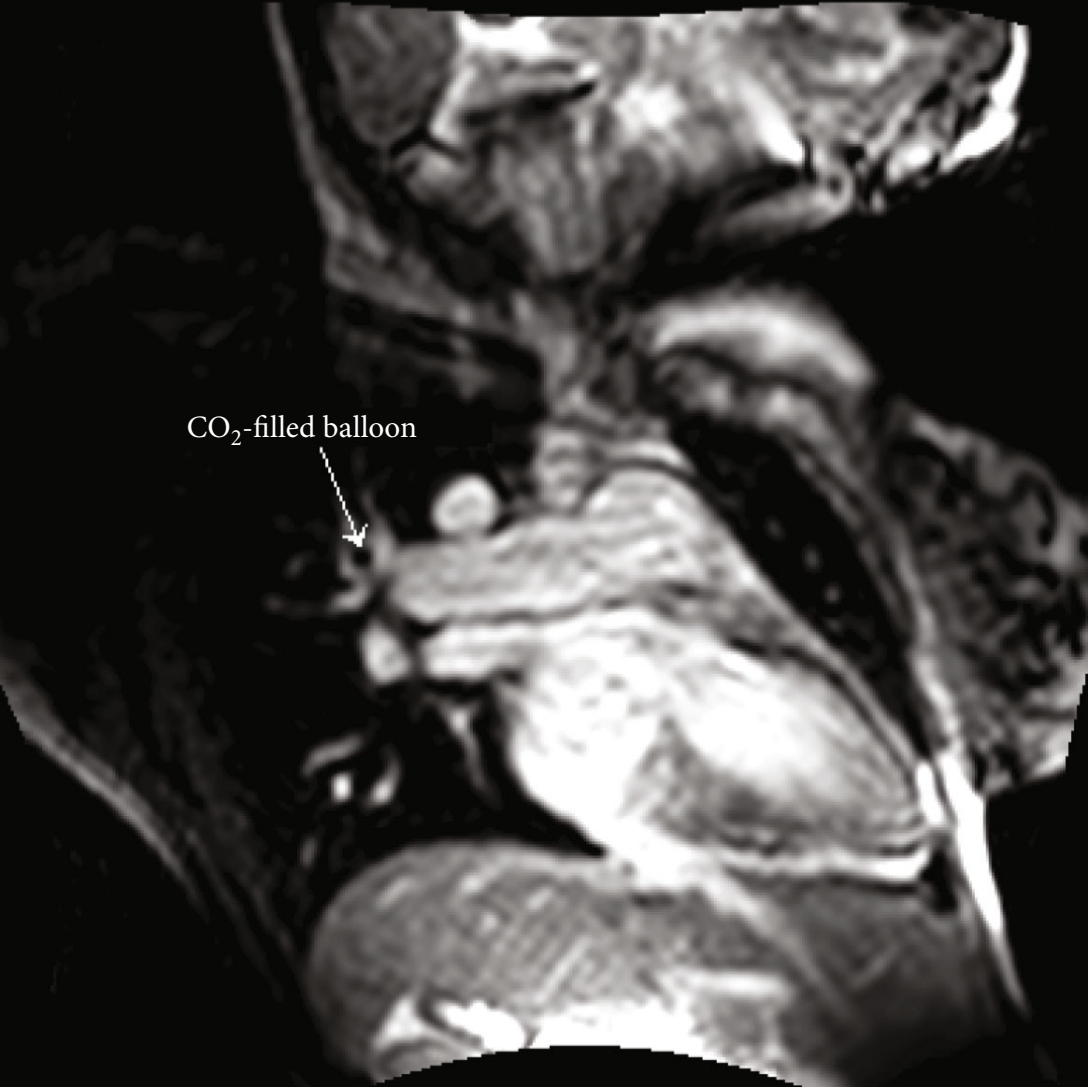
The balloon of the wedge catheter appears in the wedge position of the right pulmonary artery.

**Figure 4:**
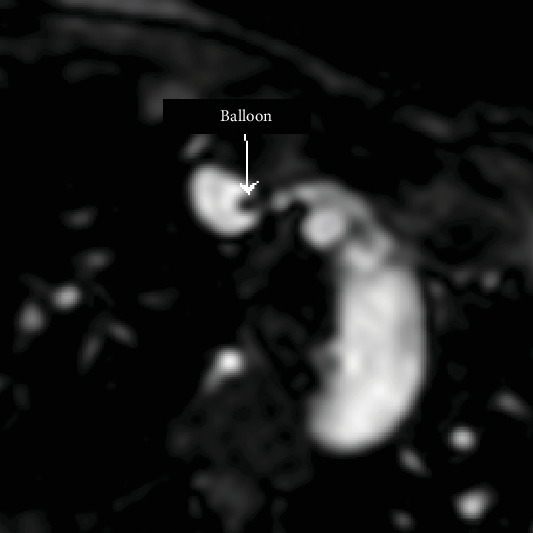
At the end of the right heart catheterization, the CO_2_-filled balloon was left inflated and the wedge catheter was left in the main pulmonary artery for simultaneous pressure measurement whilst the pulmonary flow was obtained with the phase contrast flow sequence.

Under magnetic resonance guidance, a CO_2_-filled balloon wedge catheter was inserted through the internal jugular vein without a guidewire, and the positioning of the balloon was confirmed on MRI as a black spot in the white blood pool (Figures 1 and 2). The imaging planes that were used to monitor the balloon position were the 4 chamber views, the right vertical long axis, right ventricular outflow tract, main pulmonary artery (MPA) bifurcation, and right pulmonary artery coronal views. Simultaneous recordings of the pressure waveforms in the right heart were performed. After obtaining the haemodynamic data in the MPA, the branches, and the wedge position (Figure 3), the wedge catheter was left in the MPA (Figure 4) and the pulmonary flow (Qp) was estimated using the phase contrast flow sequence. MPA pressure measured 93/29/52 mmHg against systemic pressure of 96/65 mmHg, whilst wedge pressure measured 20 mmHg. Right heart cardiac output, as estimated from the volumetric analysis of the right ventricle, was 15.5 l/min, which coincided with Qp of 15 l/min as measured by the pulmonary artery through plane flows (PA-TP) during phase contrast sequences. In comparison, LV stroke volume on the volumetric analysis was measured at 6 l/min, which coincided with Qs of 6 l/min as measured by the aortic artery through plane flows (Ao-TP) during phase contrast sequences. The estimated Qp : Qs was 2.6 : 1 with PVR of 3.3 WU·m^2^. The patient was therefore diagnosed with high pulmonary flow but sufficiently low pulmonary vascular resistance to allow her to carry on with pregnancy and plan for transcatheter closure of her atrial septal defect at a later stage.

Indeed, she carried on with pregnancy and delivered a healthy child by planned caesarean section at term. Her pulmonary arterial pressures as estimated by the TR Dopplers and oxygen saturation remained unchanged throughout pregnancy and postpartum. She received low-weight heparin during the last trimester and was converted back to aspirin after delivery. She did not suffer from any postpartum haemorrhage.

The patient was started on Sildenafil 10 mg ×2 and Macitentan 10 mg ×1 after delivery, but Sildenafil was discontinued due to nausea and hot flushes. She therefore continued with Macitentan monotherapy. She underwent repeat cardiac catheterization 2.5 years later in order to assess her PVR and suitability for transcatheter closure of the atrial septal defect. Main pulmonary artery pressures measured 72/16/40 mmHg with wedge pressure of 16 mmHg. Test balloon occlusion of the atrial septal defect was performed, which showed stable systemic pressures without increase of the venous pressures. Repeat PA pressure with the balloon occluding the defect reduced to 55/20/28 mmHg, suggesting significant flow-related element for the increased pulmonary artery pressures, as demonstrated previously during MRI-guided catheterization. PVRi was measured as 6 WU·m^2^ at baseline and dropped to 4 WU·m^2^ after reversibility testing. We therefore proceeded with closure of the defect with a 27 mm Occlutech device with a 6 mm fenestration.

At the age of 37 years, 4 years after the initial MRI-guided catheterization and 1.5 years after the fenestrated device closure, she presented for a repeat cardiac assessment in order to quantify the risk for another pregnancy. Once again, she underwent a hybrid cardiac catheterization combined with MRI assessment. Main pulmonary artery pressure at baseline measured 48/11/31 mmHg with wedge pressure of 15 mmHg, Qp : Qs of 1.22 : 1, and PVRi of 4.1 WU·m^2^, all based on MRI flows and crosschecked with catheterization data. With FiO_2_ of 100% and 20 ppm NO, repeat MPA pressures measured 43/10/28 mmHg and Qp : Qs remained at 1.2 : 1, accounting for a drop in PVRi to 3.4 WU·m^2^. The repeat hybrid catheterization procedure demonstrated a small decrease of the MPA pressure and unchanged PVR after the fenestrated device closure.

## 3. Discussion

Adults with congenital heart disease are an ever-growing population. It is estimated that in the United States, there are more adults with congenital heart disease than infants and children with the disease. Approximately half of the grown-up CHD population are women, most of whom are of childbearing age [2]. Cardiac catheterization procedures are often required, particularly when there is suspicion of pulmonary hypertension. However, this may not be feasible in pregnant women within the first few weeks of pregnancy, due to the deleterious effects of ionizing radiation on the fetus during organogenesis. We report on the technique of performing a right heart catheterization under sole MRI guidance in a pregnant woman with severe pulmonary hypertension and CHD. Solely MRI-guided diagnostic catheterizations were first introduced in children with congenital heart disease in 2003 [3], whilst MR-guided interventions were made possible by the authors' group in 2010 [4]. Performing a cardiac catheterization under MRI is quite different from fluoroscopy. MRI-compatible monitoring equipment is required, and it is essential for the monitoring equipment to have 2 ports, where invasive blood pressure measurements can be transduced from the arterial and the venous system. Although pressure monitoring can be done smoothly with the use of a balloon catheter, this type of procedure would not provide accurate flow measurements and PVR, as well as the anatomical and structural details that were required for our congenital heart disease patient.

To this end, we decided to apply a solely MRI-guided catheterization procedure. She tolerated pregnancy extremely well due to the large ASD and the ability to offload the right heart.

We watched her carefully throughout pregnancy, due to the known fact that pregnancy in women with haemodynamically significant congenital heart disease can be poorly tolerated due to the increase in blood volume and cardiac output. In this patient group, who also has pulmonary hypertension, the maternal mortality is 30-50% in older series and 9-33% in more recent papers, placing them in a high-risk category [5–7]. As pulmonary vascular disease is considered to be the worst risk factor for pregnancy and the postpartum period [8] with a 40% risk for maternal death or heart failure, despite the recent advances in pre- and postpartum care for these patients [7], assessment of pulmonary artery pressures and vascular resistance via cardiac catheterization is essential for risk stratification and appropriate consultation and therapy (class of recommendation I, level of evidence C) [9]. Traditionally, fluoroscopy has been used to guide catheters around the cardiac structures and vessels. In the recent years with the development of MRI hardware and software, it is possible to obtain real-time imaging of the heart in order to guide a cardiac catheterization procedure using MRI-compatible catheters, whilst also obtaining advanced imaging datasets (3D, flow quantification, and tissue characterization).

MRI-guided catheterization in our patient facilitated accurate measurement of haemodynamics and PVR, in a situation where fluoroscopy was a relative contraindication. Although the pulmonary arterial pressures were found to be at the systemic level, accurate measurement of cardiac output by volumetry and pulmonary flows established that the PVR was sufficiently low for pregnancy to be continued. This case depicts the various and important applications of MRI-guided catheterization that can offer unique anatomic as well as physiologic information in cases where ionizing radiation is best to be avoided.

## 4. Conclusion

The adoption of MRI-guided catheterizations for patients with CHD, who undergo multiple tests involving ionizing radiation throughout their lifetime, is a promising and safe alternative in terms of limiting radiation exposure. As the effects of ionizing radiation are increasingly deleterious with younger age, fetuses are the most vulnerable population. To this end, MRI-guided catheterization could provide a good alternative for pregnant women with cardiovascular disease who have to undergo invasive haemodynamic evaluation.

## Data Availability

The patient's file data used to support the findings of this case are restricted by the Mitera Hospital's ethic committee in order to protect patient privacy. Data are available from Dr. Aphrodite Tzifa, Department of Congenital Heart Disease, Mitera Children's Hospital, Athens, Greece, atzifa@mitera.gr, for researchers who meet the criteria for access to confidential data.

## Abbreviations

PH: Pulmonary hypertension
MRI: Magnetic resonance imaging
CHD: Congenital heart disease
PAH: Pulmonary arterial hypertension
PVR: Pulmonary vascular resistance
PVRi: Indexed pulmonary vascular resistance
MPA: Main pulmonary artery
IVC: Inferior vena cava
TR: Tricuspid regurgitation
NO: Nitric oxide.
